# Health-Promoting Properties: Anti-Inflammatory and Anticancer Properties of *Sambucus nigra* L. Flowers and Fruits

**DOI:** 10.3390/molecules28176235

**Published:** 2023-08-24

**Authors:** Agnieszka Ewa Stępień, Julia Trojniak, Jacek Tabarkiewicz

**Affiliations:** 1 Institute of Health Sciences, College of Medical Sciences, University of Rzeszow, 35-959 Rzeszów, Poland; 2 Student’s Scientific Club Immunology, Institute of Medical Sciences, College of Medical Sciences, University of Rzeszow, 35-959 Rzeszów, Poland; juliatrojniak0@gmail.com; 3 Institute of Medical Sciences, College of Medical Sciences, University of Rzeszow, 35-959 Rzeszów, Poland; jtabarkiewicz@ur.edu.pl

**Keywords:** *Sambucus nigra* L., anti-inflammatory, anticancer, antioxidants

## Abstract

*Sambucus nigra* L. has been used for centuries in traditional medicine thanks to its valuable healing properties. The healing properties result from its high content of biologically active compounds, mainly antioxidants, which contribute to its anti-inflammatory and anticancer properties. In our review, we have presented scientific studies evaluating the anti-inflammatory and anticancer effects of extracts and their components from *S. nigra* L. flowers and fruits. The results of the research show that the effect of antioxidant phytochemicals contained in their composition reduces the level of free radicals and pro-inflammatory cytokines, prevents mutations that increase the risk of cancer development, and inhibits cell proliferation, induction of apoptosis, and changes in intracellular signaling, consequently inhibiting the growth of malignant tumors and the formation of metastases. Flowers and fruits of *S. nigra* L. are a valuable source of nutraceutical and pharmacological substances that can support prevention and anti-inflammatory and oncological therapy without negative side effects for the patient.

## 1. Introduction

In recent years, we have observed a trend in scientific research to return to the assessment of the medicinal properties of plants that are the basis of traditional medicine. Plants are a rich source of biologically active compounds defined as phytochemicals, which are used in the prevention and treatment of many diseases. Bioactive substances present in plants and secondary metabolites are mainly antioxidants with antioxidant properties. Numerous processes in the body generate free radicals, like reactive oxygen or nitrogen species, that are neutralized by endogenous compounds with antioxidative properties. However, a persistently high level of unneutralized free radicals in the body can cause the induction of oxidative stress, which increases the risk of inflammation [1,2].

The inflammation associated with infections, e.g., viral or bacterial infections, plays an important role in the development of carcinogenesis [3]. Exogenous antioxidants present in the diet significantly counteract the development of oxidative stress and decrease the risk of chronic inflammation, leading to cancer [4].

Among medicinal plants, *Sambucus nigra* L. draws special attention as a rich source of many bioactive compounds, such as polyphenols and flavonoids, with antioxidant properties.

*Sambucus nigra* L. (*S. nigra* L., *S.nigra)*, also called European wild black elder, elderberry, European elderberry, European elder and European black elderberry, belongs to the family *Adoxaceae* Trautv., i.e., *Viburnaceae* Raf. from the order *Dipsacales* and earlier, and also to *Caprifoliaceae* Juss. and *Sambucaceae* Batsch [5,6,7].

**Figure 1:**
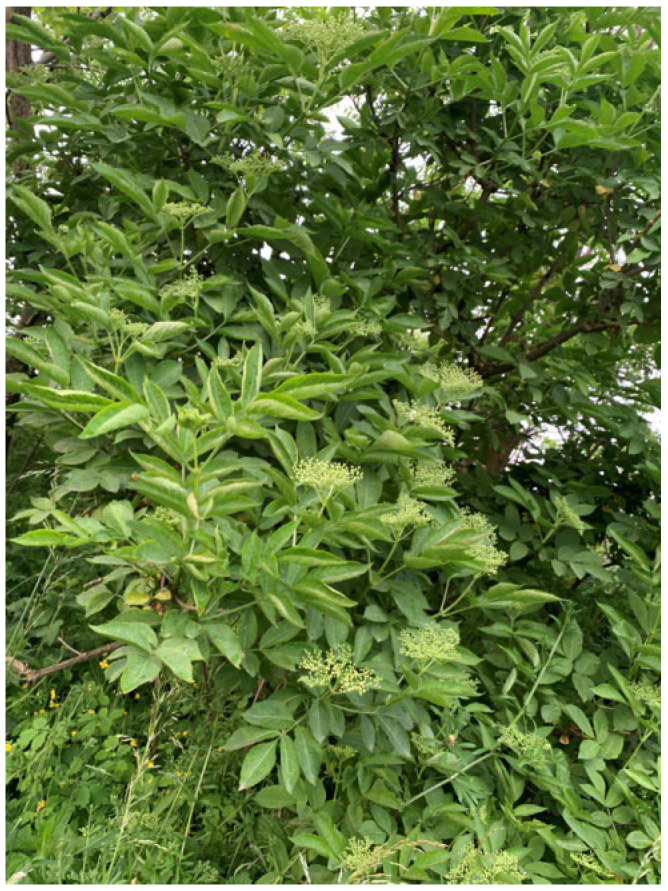
The *S. nigra* L. bush (photograph by Julia Trojniak).

*S. nigra* L. is found in natural habitats in Asia, North America, Europe, North Africa, New Zealand and Australia as a small deciduous tree or shrub, usually 4 to 12 m tall. *S. nigra* L. grows in the wild, usually in open fields or on forest edges. Its branches are often arching, the bark of which turns green to brownish-gray as it ages and cracks. The leaves are pinnate, arranged opposite each other, and vary in shape from ovate to ovate-lanceolate to ovate-elliptical. They usually measure from 3 to 9 cm in length with a tail (3–4 cm) [8,9] (Figure 1).

**Figure 2:**
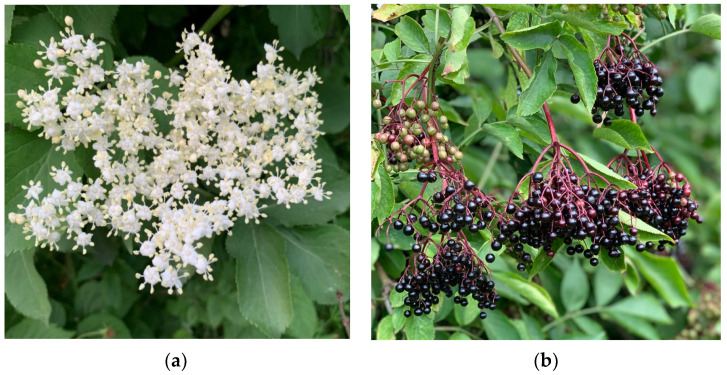
*S. nigra* L. flower (**a**) and fruits (**b**) (photograph by Julia Trojniak).

The inflorescences of *S. nigra* L. are flattened, apical, and umbellate with five main rays, with milky white flowers with five yellow stamens, and a faint and aromatic fragrance with a slightly bitter taste (Figure 2a) [10,11]. The fruit is a spherical drupe with 3–5 pressed seeds. Initially, the fruits are green, but in the process of ripening, they change color from red to black-purple with a shiny skin and fleshy with a lot of juice [9]. *S. nigra* L. blooms in May and June, while the fruits usually ripen from July/August to October (Figure 2b) [12]. For aesthetic reasons, it is planted in parks and gardens, as well as on plantations, especially to obtain pharmaceutical raw materials.

All raw parts of *S. nigra* L. are poisonous due to the presence of cyanogenic glycosides (CNGs), including the most abundant sambunigrins and prunazines. These compounds, in the presence of β-glucosidase and α-hydroxynitrile lyase enzymes, release highly toxic hydrogen cyanide (prussic acid, HCN) [13]. Elderberry leaves contain more sambunigrin than the flowers and the lowest amount of this compound is in the berries. It was also found that the content of sambunigrin in elderberries varies depending on the height of cultivation. It is necessary for it to undergo thermal treatment, which causes the decomposition of sambunigrin to compounds harmless to the human body [14]. In addition to poisonous compounds, *S. nigra* L. is also rich in nutrients, such as carbohydrates, proteins, fats, fatty acids, organic acids, minerals, vitamins and essential oils [14]. The chemical composition of individual morphological parts of the plant depends on many factors, including environmental conditions, such as soil type, light, temperature, amount and frequency of rainfall, fertilization, cultivation methods and processing and storage conditions, as well as altitude above sea level [15].

For centuries, black elderberry flowers and fruits have been used in the kitchen as an addition to cakes and in the preparation of jams and tinctures due to their taste.

The results of the bioavailability and safety assessment of *S. nigra* L. flowers and fruits showed no toxic effects or side effects on the human body. Flowers and fruits of *S. nigra* L. are recognized by the US Food and Drug Administration (FDA) as a safe food additive, which is indicated by the GRAS (Generally Recognized As Safe) status. The European Medical Agency (EMA) points to the safety of their use but recommends limiting their use by women during pregnancy and lactation as well as in children under 12 years of age [16,17].

*S. nigra* L., for a long time, has been one of the most frequently used plants in traditional medicine for the treatment of many diseases with diaphoretic, anti-inflammatory, diuretic or antipyretic effects [18,19]. *S. nigra* L. has antioxidant, anti-inflammatory, cytotoxic, anti-allergic, immunomodulating and antiviral, antibacterial, antidepressant, antidiabetic, antiatherosclerotic and hypoglycaemic properties [6,8,19,20]. It also shows anticancer activity, which determines its supporting role in the prevention and treatment of cancers with limited side effects in this form of therapy. All parts of the S. *nigra* L. plant, flowers, leaves, fruits and bark contain numerous bioactive compounds—mainly with antioxidant properties—that determine its health-promoting properties, making them a very valuable pharmaceutical raw material.

Oxidative stress is the result of an imbalance of free radicals-antioxidants in the body as a result of excessive amounts of free radicals (reactive oxygen species (ROS) and nitrogen). Long-term oxidative stress associated with chronic inflammation can lead to the development of many diseases. Reduction of oxidative stress is possible by increasing the level of antioxidants in the body. This indicates the very important role of the presence of vegetables, fruits or medical plants with a high content of polyphenols in the diet because, thanks to the neutralization of free radicals, i.e., reactive oxygen species, they protect cells and tissues against their negative effects, influence the induction of antioxidant enzymes and strengthen the immune response [21].

Flowers (*Sambuci Flos*) and fruits (*Sambuci Fructus*) are the richest in polyphenolic compounds, mainly flavonols, phenolic acids and anthocyanins, which affect their properties see Table 1 and Table 2. The results of a previous study showed the high antioxidant capacity of elderberries—an average of 4.31 mmol/Trolox equivalent (TE) [22]. Such high antioxidant activity results from the high content of antioxidants, mainly phenolic compounds, but also minerals, vitamins C and E, and carotenoids that affect these abilities [22].

This article presents the results of scientific research determining the health-promoting properties of *S. nigra* L. flowers and fruits, including both anti-inflammatory and anticancer properties. The literature review describes current scientific research focusing on important aspects, such as phytochemical analysis, evaluation of the pharmacological aspect of such action of *S. nigra* L. flower and fruit extracts and phytochemicals present in their composition.

## 2. Anti-Inflammatory Properties of *S. nigra* L.

Inflammation is a natural protective mechanism of the body, preventing damage to its tissues. The mechanism of the inflammatory process is based on the activation of monocytes and/or macrophages present in various tissues that determine its course through the release of inflammatory cytokines such as interleukins (IL) and tumor necrosis factor (TNF-α) and inflammatory mediators such as ROS and nitric oxide (NO), or prostaglandin E2 and cyclooxygenases (COX) and lipoxygenases (LOX) [27]. It was determined that the main role in the development and progression of inflammation and the immune response is played by the activity of regulatory enzymes, such as lipoxygenases and cyclooxygenases. Cyclooxygenase mediates the development and regulation of the inflammatory process through the synthesis of eicosanoids (prostaglandins and prostacyclins) via the arachidonic acid pathway. The increased synthesis of prostaglandins is also affected by the accelerated synthesis of nitric oxide by nitric oxide synthase in the area of inflammation [28].

Currently used therapies are based on drugs acting to suppress the pro-inflammatory response; however, these have limited effectiveness, and importantly, some of them have undesirable health effects for patients. In recent years, the role of preparations based on plant materials, mainly medicinal plants rich in numerous bioactive compounds that do not have side effects for patients, has been focused on in the prevention and therapy of diseases associated with inflammation.

Ho et al. analyzed the effect of *S. nigra* L. fruit and flower extracts and various polyphenols present in these extracts on lipopolysaccharide (LPS)-stimulated RAW 264.7 macrophages. The juice and the acidified methanol extract, both at a concentration of 100 µg/mL, inhibited the secretion of pro-inflammatory factors by macrophages by 30% and 50%, respectively. Among polyphenolic compounds, cyanidins, cyanidin-3-glucoside and cyanidin-3-glucoside sambubioside (100 µM) were reduced by about 60% to 70%, as well as quercetin by 80% (100 µM), and among phenolic acids, chlorogenic acid by about 51.5%. All these compounds are present in elderberry extracts, and their high content in them contributes to the high anti-inflammatory effect of the extracts [29].

The anti-inflammatory properties of quercetin result from its influence on the inhibition of the secretion of pro-inflammatory cytokines such as TNF-α and interleukins (IL-1β, IL-6) and the reduction of cyclooxygenase and lipoxygenase activity [30,31]. In an in vivo study, the administration of quercetin to diabetic rats also reduced the level of prostaglandins and interleukins (IL-1β) [32]. Incubation of quercetin with an LPS-stimulated macrophage line (RAW264.7) resulted in the inhibition of TNF-α generation and nitric oxide synthase activity [33]. It has been shown that it also affects the production of anti-inflammatory cytokines such as IL-10 [34].

In subsequent in vitro studies, the antioxidant and anti-inflammatory effects of *S. nigra* L. fruit extracts were also assessed using LPS-stimulated macrophages (RAW 264.7). An inhibitory effect on the release of pro-inflammatory factors was also observed, confirming the anti-inflammatory effect. Human hepatoma (hepG2) and human colon adenocarcinoma (Caco-2) cells were treated with cytotoxic tert-butyl hydroperoxide, and then *S. nigra* L. extract was added. It inhibited the release of reactive oxygen species as a result of the cytotoxic effect of tert-butyl hydroperoxide in the case of both cell lines, preventing their damage caused by oxidative stress. The high antioxidant and anti-inflammatory activity of *S. nigra* L. fruit extract results from its phytochemical profile, confirming the presence of antioxidants [35].

Santin et al. studied the effect of *S. nigra* L. flower extract on inflammation in vivo and in vitro. They orally administered *S. nigra* L. extract at 30, 100, 300 or 600 mg/kg, respectively, to groups of male mice with carrageenan-induced inflammation. They observed that it affected the inhibition of neutrophil migration and the reduction of the level of the pro-inflammatory cytokines TNF, IL-1β and IL-6. In vitro, neutrophils and macrophages stimulated with lipopolysaccharide were also treated with this extract at concentrations of 1, 10 or 100 μg/mL. They showed a decrease in the levels of reactive nitrogen forms in neutrophils and the pro-inflammatory cytokines TNF, IL-1β and IL-6, and an increase in anti-inflammatory IL-10 and neutralized expression of CD62L and CD18. Researchers point to the role of rutin, the main component of *S. nigra* L. extract, in such an effect. In macrophages, the effect is comparable in reducing NO_2_, TNF and IL-6. Extracts from *S. nigra* L. flowers show anti-inflammatory activity by modifying the secretion of pro-inflammatory cytokines by macrophages and neutrophils, which significantly affects the treatment of acute inflammation, confirming the medicinal properties of this plant [36].

The research results indicate that the anti-inflammatory effect of rutin resulting from its antioxidant properties is based on the reduction of the concentration of the pro-inflammatory markers tumor necrosis factor-α, interleukin (IL)-6, cyclooxygenase-2, and IL-1β [37].

In vitro studies have shown that kaempferol, by inhibiting the activity of COX1 and COX2 enzymes, prevents the development of inflammation. This is indicated by the results of in vitro studies where incubation of kaempferol with human hepatocytes decreased COX2 levels [38]. Subsequent studies indicate that kaempferol incubated with macrophage cells (J77, RAW264.7) stimulated with LPS significantly influenced the synthesis of nitric oxide. By inhibiting the synthesis of nitric oxide, it reduced inflammation. In vivo studies have shown that the inclusion of kaempferol in the treatment of patients with diabetic neuropathy reduced the amount of IL-1B, TNF-α and nitric oxide [39,40]. It was observed that kaempferol in diabetic nephropathy had a significant effect on the suppression of inflammation [41].

**Figure 3:**
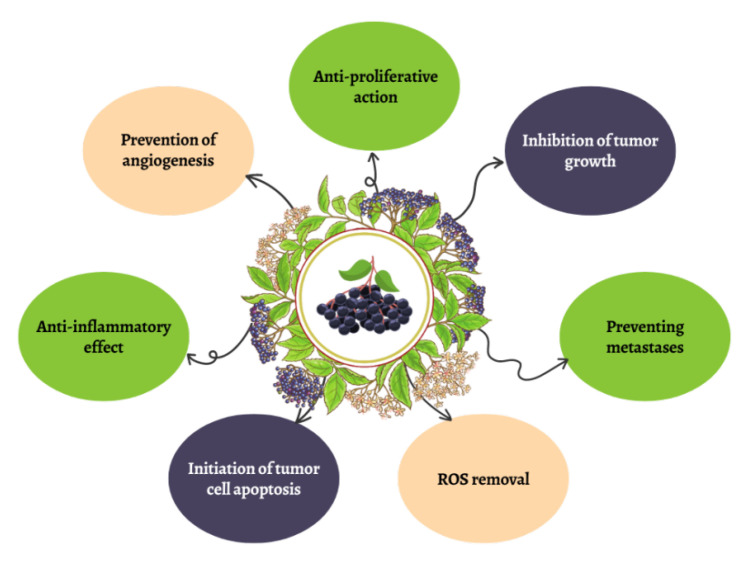
Selected properties of *S.nigra* L.

The results of the above studies show the ability of the extract and its components of elderberry fruit and flowers to protect against the development of oxidative stress and inflammation (Figure 3).

## 3. Anticancerogenic Properties of *S. nigra* L.

Worldwide, cancer is the second leading cause of death and more and more new cases of malignancy are being diagnosed, placing a significant burden on healthcare systems to provide full care to cancer patients. Current forms of cancer therapy are often accompanied by numerous negative side effects, which additionally affect the deterioration of the patient’s health. For several years, scientists have been focusing on conducting research to develop new, effective methods supporting anticancer therapy as well as reducing side effects.

The causes of cancer development are complex, and both intrinsic and extrinsic factors contribute to this complex process. The consequences of the impact of emerging free radicals, e.g., reactive oxygen species in the human body, are one of the main etiological factors of cancer [3]. Free radicals attacking cell components may contribute to damage to cell structures, and oxidative DNA damage is the cause of mutations in genes or chromosomes, leading to the development of a multi-stage carcinogenesis process [1]. Chronic inflammation as a result of oxidative stress within the cells of a given organ, resulting from the activity of excess free radicals in the human body, plays an important role in the development of carcinogenesis [3]. It is, therefore, important to increase the supply of exogenous antioxidants in the diet that counteract the development of oxidative stress, significantly affecting the deterioration of human health [4].

*S. nigra* L. contains bioactive substances and secondary metabolites, including antioxidants, that can counteract the negative role of oxidative stress, which increases the risk of cancer development [2]. It exhibits such an anticancer effect due to its influence on, among others, inhibition of cancer cell proliferation and stimulation of immune cells [42].

Flowers and fruits of *S. nigra* L. are a rich source of many biologically active compounds, mainly antioxidants that determine anticancer properties—see Figure 3.

Elderberry flowers are particularly rich in bioactive compounds with antioxidant properties, mainly flavonoids such as kaempferol, quercetin and rutin, phenolic acids and their glycosides, triterpenes and sterols—see Table 1.

In their in vitro study, Periera et al. indicated cytotoxic effects of *S. nigra* L. flower extracts on human bladder carcinoma T24 cells. The UPLC-DAD-MS/MS analysis of the chemical composition of the butane fraction of the extract allowed the identification of nine flavonoids, including rutin, quercetin and kaempferol. The obtained results indicate that flavonoids are probably responsible for the cytotoxic effect on cells of this type of cancer [10]. 

It has also been shown that the alcoholic extract of elderberry flowers affects the proliferation of a breast cancer cell line (MCF7). The results indicate that incubation of MCF7 cells with different concentrations of this extract significantly affected the level of ERα and PR steroid receptors in these cells. A decrease in ERα and an increase in PR were observed by immunocytochemistry [43].

This indicates the important role of the ingredients present in the *S. nigra* L. flower extract in chemoprevention and/or cancer therapy. Scientists undertook further research to assess the anticancer effect of compounds present in the extract of *Sambucus nigra* L. flowers, especially flavonoids [44].

**Figure 4:**
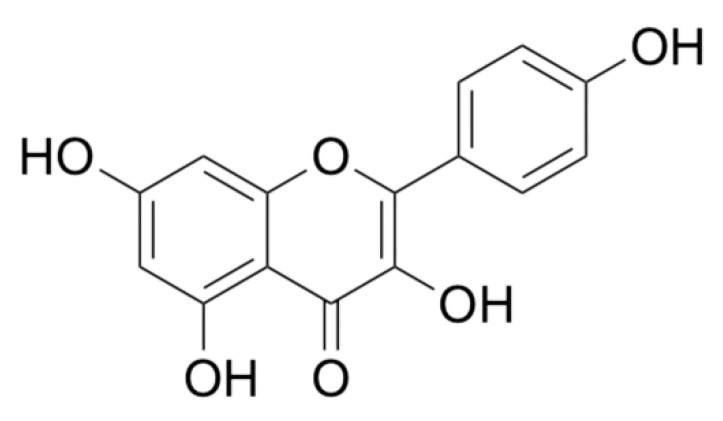
Chemical structure of kaempferol.

Scientific research indicates the high antioxidant potential of kaempferol from the group of flavonoids due to the capture of free radicals, mainly reactive oxygen species, which determines its anticancer properties—see Figure 4. Wang et al., in their in vitro study, evaluated the effect of kaempferol on human pancreatic cancer cell lines (MIA PaCa-2 and PANC-1). Thanks to the method based on the CCK-8 tests, the proliferation of cancer cells was analyzed, and the level of ROS and apoptosis of these cells were analyzed by flow cytometry. It was observed that the viability of PANC-1 and MIA PaCa-2 malignant cells decreases in the kaempferol in a dose-dependent manner. However, the results of additional in vivo studies on the effect of kaempferol on mice with pancreatic cancer indicate that tumor weight and volume decreased significantly, depending on the level of ROS. The anticancer properties of kaempferol result from its ability to induce apoptosis of cancer cells dependent on the regulation of ROS levels [45].

Subsequent in vitro studies also confirm the cytotoxic properties of kaempferol. Pham et al., using the MTT test (3-(4,5-dimethylthiazol-2-yl)-2,5-diphenyltetrazolium bromide), analyzed the viability of cancer cells after incubation with kaempferol. They determined its very strong inhibitory effect on the viability of the following human cancer cell lines: ovarian (A2780), lung (H460), skin (A431), pancreas (MIA PaCa-2), prostate (DU145), colon adenocarcinoma (HT29), breast (MCF-7), neuroblastoma (BE2-C) and glioblastoma (U87) [46]. In their in vitro studies, Akram et al. also indicated that kaempferol can arrest the cell cycle of human breast cancer cells (MDA-MB-453) [47]. The results of other studies indicate that the mechanism of action of kaempferol on human breast cancer cells (MDA-MB-231 and BT-474) is mainly the effect on inhibiting the growth of these cells, inducing their apoptosis and inhibiting their migration [48,49]. *Sambuci flos* also contains quercetin, which, thanks to its ability to neutralize free radicals—mainly reactive oxygen species—and to bind to transition metal ions, has chemopreventive properties [50,51,52]. The analysis of the results of in vitro studies on cancer cell lines indicates the possible anticancer effect of quercetin in relation to various types of cancer, such as ovarian cancer, breast cancer, colorectal cancer and prostate cancer [53,54,55,56]. Khan et al. indicate that among the phytochemicals, quercetin may be an alternative therapeutic option with limited side effects in the treatment of ovarian cancer. This emphasizes the interplay between it and miRNA in the regulation of apoptosis in this type of cancer [53]. Khorsandi et al. studied the effect of quercetin on the growth of a human breast cancer cell line (MCF-7) and indicated its high toxicity to cells of this type of cancer. They also determined that the mechanism of MCF-7 cell death as a result of the action of quercetin involves many pathways, mainly necroptosis [54].

In vivo studies in an animal model with colorectal cancer have evaluated the anticancer properties of quercetin. The presence of quercetin in the diet of rats treated with the carcinogenic substance methane nitroxin significantly influenced the development of the disease. Compared to the control and healthy group, it was shown that the presence of quercetin (10 mg/kg of body weight) in the diet reduces cytological and morphological changes in the cells of healthy rats. This indicates the possibility of its use in the prevention and treatment of colon cancer [55].

Quercetin, a bioflavonoid, also has anticancer effects against prostate cancer. Incubation of human prostate carcinoma cells (DU-145 and PC-3, LNCaP) with quercetin significantly improved their viability. A decrease in tumor cell viability was observed compared to the control, with no effect on healthy prostate epithelial cells, depending on the quercetin concentration and incubation time. The authors emphasize that quercetin exerts anticancer effects by modulating the ROS, Akt and NF-κB pathways. They also indicate that it can be used as a chemopreventive option, as well as in combination with chemotherapeutic drugs, to improve the clinical outcomes of patients with prostate cancer [56].

Research has been undertaken to determine the cytotoxic activity of compounds from the group of flavonoids quercetin and rutin. Both compounds have been shown to inhibit the growth of leukemic cells (HL-60) in a dose-dependent manner [57].

**Figure 5:**
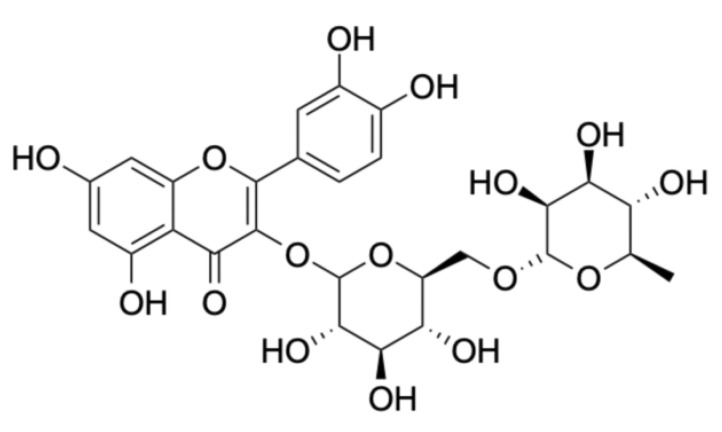
Chemical structure of rutin.

Rutin (quercetin-3-rutinoside), from the group of flavonoid glycosides, is present in *Sambucus nigra* L. flowers—see Figure 5. It is characterized by antioxidant properties, inhibition of lipid peroxidation by reducing COX-2 expression, reducing the level of oxidative stress and inducing the activity of nitric oxide synthase (iNOS), as well as exhibiting anti-inflammatory and anticancer properties [58,59,60].

Satari et al. undertook a study to evaluate the effect of the joint action of rutin and the anticancer drug 5-fluorouracil on the human prostate cancer cell line (PC3). In previous studies, they showed that rutin induces apoptosis of prostate cancer cells [58]. They determined cell viability, *p53* gene expression, Bcl-2 signaling protein changes and apoptosis. The results of the studies indicate the synergistic effect of this drug and rutin on the apoptosis of cells of this type of cancer [59]. Subsequent studies confirm the anticancer properties of rutin. It was determined that the incubation of the human neuroblastoma cell line (LAN-5) with rutin had a significant effect on the viability of these cells and induction of G2/M arrest in the cell cycle [60]. Rutin also has a valuable antioxidant, antibacterial, anti-inflammatory, and UV-filtering effect on the skin. Its cytotoxic properties against two human melanoma cell lines (RPMI-7951 and SK-MEL-28) were evaluated. A concentration-dependent decrease in the viability of cancer cell lines was demonstrated, indicating its role in inducing apoptosis of cancer cells by increasing the expression of beta-galactosidase associated with aging (SA-β-gal) [61].

The results of the above studies emphasize the chemotherapeutic role of the natural compound rutin in the prevention and treatment of various types of cancer.

**Figure 6:**
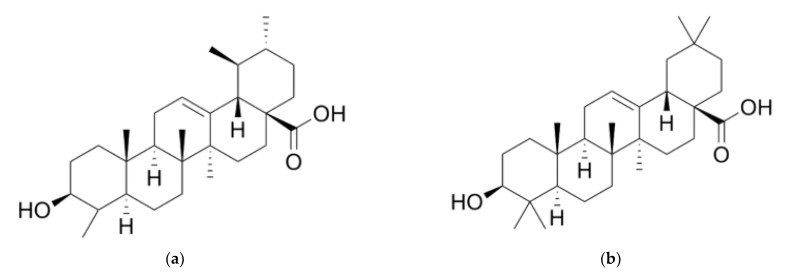
Chemical structure of triterpene compounds: (**a**) ursolic acid (C_30_H_48_O_3_); and (**b**) oleanolic acid (C_30_H_48_O_3_).

Ursolic and oleanolic acids are common triterpenoid compounds found in *S. nigra* L. flowers (Figure 6). Due to their antioxidant properties, these acids exhibit various pharmacological effects. The potential use of these triterpenoids in anticancer therapies, including treatment, as well as their impact on the immune system, is also indicated [62].

Yang et al., in their research, analyzed the effect of oleanolic and ursolic acids on human liver cancer cell lines (HepG2, Hep3B, Huh7 and HA22T). They observed that both acids significantly reduced the viability of these cells by inducing apoptosis [63].

Studies have shown that ursolic acid may have beneficial effects in various types of cancers, including those caused by inflammation.

The results of a previous study indicate that incubation of ursolic acid with human breast cancer cell lines (MCF-7) inhibits cell proliferation and induces apoptosis, depending on time and concentration [64].

It is important to understand the mechanisms of anticancer activity of ursolic acid at the molecular level. Wang et al., in their in vitro studies, determined the molecular mechanisms of action of ursolic acid on human colon cancer cells (SW480, LoVo). Incubation of these cell lines with ursolic acid significantly inhibited their viability as well as their ability to migrate. The influence of ursolic acid on the signaling pathways MMP9/CDH1, Akt/ERK, COX-2/PGE2 and p300/NF-κB/CREB2 and cytochrome c/caspase pathways of colorectal cancer cells determines its anticancer properties [62].

The results of subsequent in vitro studies indicate the anticancer properties of ursolic acid on breast cancer cells (T47D, MCF-7 and MDA-MB-231). It has been observed that it induces autophagy and apoptosis of these cells through the GSK and Bcl-2/caspase-3 signaling pathways and also prevents the development of inflammation. In contrast, the mechanism of inhibition of breast cancer cell proliferation by ursolic acid is based on the inactivation of the PI3K/AKT signaling pathway. Understanding the mechanism at the molecular level of the effect of this acid on the cells of this type of cancer determines its possible use in breast cancer therapy [65]. Another important element of the anticancer activity of ursolic acid is the effect on the CXCR4/CXCL12 signaling pathway, which is important in the development of metastases. The effect of ursolic acid reduces the expression of CXCR4 by regulating the transcription of mRNA expression and inhibiting the activation of NF-κB [66]. The results of numerous in vitro studies indicate that it activates the growth inhibition of these cell lines due to changes in signaling pathways at the molecular level; in vivo studies also show its cytotoxic effects against these types of cancer. The results of research indicate that it affects numerous pro-inflammatory factors, including cytokines, cell cycle proteins, inflammatory enzymes, growth factors and kinases. It is indicated that by inhibiting the development, growth and metastasis of cancer, it determines its potentially chemopreventive and therapeutic effect [67].

*S. nigra* L. flowers contain the most phenolic compounds compared to fruits and leaves, which results in the highest antioxidant activity among all parts of this plant [14,18].

**Figure 7:**
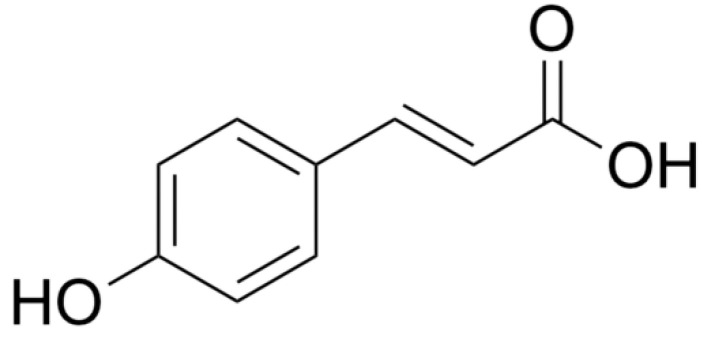
Chemical structure of p-coumaric acid.

The group of phenolic acids present in elderberry flowers includes p-coumaric acid (p-CouA), which is a derivative of cinnamic acid [68] (Figure 7). Scientific research indicates its high biological activity, such as antioxidant properties, the ability to neutralize free radicals, e.g., reactive oxygen species, and anti-inflammatory properties [69]. This affects its anticancer properties, i.e., the ability to induce apoptosis and block the cell cycle, which inhibits their growth [68]. Its cytotoxic effect has been demonstrated against many cancer cell lines. Effects have been observed in vitro on the growth of neuroblastoma (N2a), human lung (A549), colon (HT29-D4) and cancer stem cells, colon cancer (Caco-2), the human endothelial cell line (ECV304), breast cancer (MCF7), and liver cancer cell lines (HPG2) that significantly reduce the viability of these cells [70].

**Figure 8:**
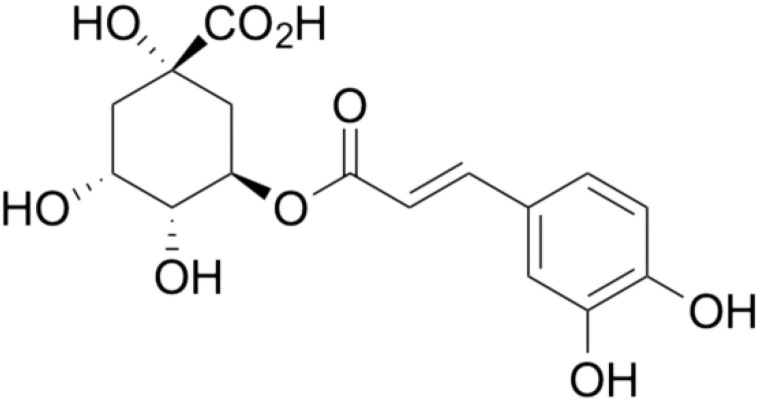
Chemical structure of chlorogenic acid.

The phenolic acid present in the flower of *S. nigra* L. is also chlorogenic acid—see Figure 8. Its antioxidant properties determine its healing effect, including its anticancer effect.

The results of research showed that chlorogenic acid affects proliferation and inhibits angiogenesis and the growth of metastases of cancer cells. Its cytotoxic activity against breast cancer cells (MCF-7), colon cancer cells (HT-29), human hepatocarcinoma cells (HepG2), human leukemia cells (U9370) and human lung cancer cells (A549) was demonstrated in a dose- and time-dependent manner [71]. Further studies indicate that chlorogenic acid inhibits the proliferation of human lung cancer cells (A549) by inducing apoptosis [72]. Zeng et al. analyzed the effect of chlorogenic acid on human breast cancer cell lines (MDA-MB-231, MDA-MB-453 and MCF-10A) and the murine breast cancer 4T1 cell line. Incubation of the MDA-MB-231, MDA-MB-453 and 4T1W lines with chlorogenic acid resulted in the inhibition of the growth of these cells. However, its effect on human MCF-10A cells, unlike cancer cells, did not affect their viability and did not inhibit their growth. The results of the in vitro study indicate that chlorogenic acid has the ability to selectively affect the viability and inhibit the proliferation of breast cancer cells [73].

*S.nigra* L. fruits also contain numerous compounds with high biological activity, among which anthocyanins and polyphenols have the highest activity—see Table 2.

**Table 2:** Selected bioactive chemical components of *Sambuci Fructus*.

The Group of Chemicals	Examples of Substances		Contents
Carbohydrates	monosaccharides	glucose, fructose	Total: 6.8–11.5%/68.53–104.16 g/kg [14,23] 95% of total
	pectin		7.4% of total
Vitamins	ascorbic acid (vitamin C), riboflavin (vitamin B2), pyridoxine (vitamin B6), niacin (vitamin B3), pantothenic acid (vitamin B5), folic acid (vitamin B9)		Variable
Organic acids	acetic acid, malic acid, shikimic acid, valeric acid, tartaric acid, benzoic acid, ursolic acid, oleanolic acid		1.0–1.3%
Phenolic acids	Chlorogenic acid, neochlorogenic acid, cryptochlorogenic acid		371–432 mg GAE ^1^/100 g [74]
Anthocyanins	Cyanidin 3-glucoside—Figure 9a, Cyanidin 3-sambubioside—Figure 9d, Cyanidin 3-sambubioside-5-glucoside—Figure 9c, Cyanidin 3,5-diglucoside—Figure 9e, Cyanidin 3-rutinoside—Figure 9b, Pelargonidin 3-glucoside—Figure 9g, Pelargonidin 3-sambubioside—Figure 9h, Delphinidin 3-rutinoside—Figure 9f		242–283 mg CGE ^2^/100 g FW ^3^; 272.87 mg/100 g FW; 664–1816 mg CGE/100 g FW; 8.33–101.40 mg CGE/g DW ^4^; 1.9–20.2 g CGE/kg; 170–343 mg CGE/100 g; 465.1 mg/100 g FW; 602.9–1265.3 mg CGE/100 g FW; 1374.4 mg CGE/100 g [23,74]
Flavonols	Quercetin 3-O-rutinoside, Quercetin 3-O-galactoside, Quercetin 3-O-vicianoside, Quercetin 3-O-glucoside, Quercetin 3-O-(6″-acetyl)galactoside, Quercetin 3-O-(6″-acetyl)glucoside, Kaempferol 3-O-rutinoside, Kaempferol 3-O-glucoside, Isorhamnetin 3-O-rutinoside, Isorhamnetin 3-O-glucoside, Myricetin 3-O-rutinoside		38.26 mg/100 g FW; 13.6978–20.1836 g/100 g extr ^5^; 57.0–102.7 mg QRE ^6^/100 g [23]

^1^ GAE—gallic acid equivalents; ^2^ CGE—cyanidin 3-glucoside equivalents; ^3^ FW—fresh weight; ^4^ DW—dry weight; ^5^ extr.—extract; ^6^ QRE—quercetin 3-rutinoside equivalents.

In vitro studies indicate the antioxidant properties of *Sambucus nigra* fruit extracts. These compounds can also increase the activity of enzymes in the small intestine, liver and lungs, including peroxidase, S-transferase, glutathione reductase and catalase [12,75]. Studies have shown that *S. nigra* L. fruit extracts inhibit both the initiation phase and the development phase of carcinogenesis. This indicates the significant cytotoxic properties of these extracts, which inhibit the proliferation of cancer cells. These data indicate that in addition to the cytotoxic effect on cancer cells, the extracts may have a cytoprotective effect on healthy cells and enhance the immune response, supporting the body’s response to cancer [42].

Anthocyanins are the main group of fruit polyphenols, mainly cyanidin-3-glycoside, cyanidin-3-sambubioside and cyanidin-3-diglycoside [15].

The ability of anthocyanins to neutralize free radicals and reduce oxidative stress affects their anti-inflammatory and anticancer properties. Liu et al. studied the in vitro effect of cyanidin-3-glucoside on HER2-positive breast cancer cells. The results indicate that it induces apoptosis of HER2-positive breast cancer cells. In vivo studies showed that it reduced the size and volume of the tumor [76]. In addition, cyanidin-3-glucoside reduces the level of glutathione and phosphorylated MAP kinases, including ERK1/2, p38, JNK1/2 and MKK4, as well as pro-inflammatory cytokines [76]. In subsequent studies, it was determined that cyanidin-3-glucoside weakens breast cancer-induced angiogenesis by inhibiting VEGF, a key cytokine for this process [77]. Research results indicate the photochemopreventive or anticancer abilities of anthocyanins against skin cancer. Studies have been undertaken involving the effect of cyanidin-3-glucoside on the B16-F10 metastatic murine melanoma cell line. It was determined that this anthocyanin isolated from *S. nigra* L. fruit induces apoptosis of melanoma cells and inhibits their proliferation [78].

## 4. Conclusions

Flowers and fruits of *S. nigra* L. are a natural potential source of valuable nutraceutical and pharmacological substances.

The presence of biologically active substances with antioxidant properties in the flowers and fruits of *S. nigra* L., mainly polyphenols and anthocyanins, determines their health-promoting properties, e.g., anti-inflammatory and anticancer properties.

An important role in these processes is attributed to flavonoids, triterpenoid compounds and anthocyanins, as well as phenolic acids present in extracts from the flowers and fruits of *S. nigra* L. The results of scientific research indicate that these bioactive compounds inhibit the development of inflammation as a result of excessive activity of free radicals, alleviating the symptoms of diseases associated with inflammation by modifying the activity of macrophages and neutrophils and the secretion of pro-inflammatory cytokines, which significantly affects the treatment of acute inflammations, confirming the healing properties of this plant. It has been shown that *S. nigra* L. is a rich source of flavonoids, mainly kaempferol, quercetin and rutin, as well as phenolic acids, and it also inhibits many enzymes and mediators, e.g., prostaglandins and nitric oxide synthase, that cause inflammation.

Research results indicate the cytotoxic properties of extracts from flowers and fruits of *S. nigra* L. and their components—mainly rutin, quercetin, oleanolic and ursolic as well as chlorogenic and p-coumaric acids—against bladder, breast, pancreatic, skin, lung, colon, ovarian, liver, prostate and leukemia cells.

Their influence on cancer cells, metabolic pathways and the regulation of individual mechanisms of proliferation, angiogenesis, growth and induction of apoptosis indicates that *S. nigra* L. extracts can support modern oncological therapy with limited side effects. Both the antioxidant and anti-inflammatory effects of *S. nigra* L. extracts—due to capturing reactive oxygen species and, thus, preventing mutations—and their pro-apoptotic effects have a proven basis for their use in the prevention and supportive treatment of inflammatory and cancer diseases.

Further research on the molecular mechanisms of the pro-inflammatory and anticancer effects of extracts and their individual components of *S. nigra* L. flowers and fruits is necessary to determine the level of the effective dose of such a preparation supporting the prevention and therapy of various inflammatory and cancer diseases. This suggests the need for more human clinical trials. It will also make it possible to determine the level of a safe dose of such a medicine of natural origin that does not pose a threat to health or possible progression of other comorbidities in patients.

It is also important to undertake research to assess the interaction between the phytochemicals present in the flowers and fruits of *S. nigra* L. with other drugs used in anticancer therapy.

## Figures and Tables

**Figure 9:**
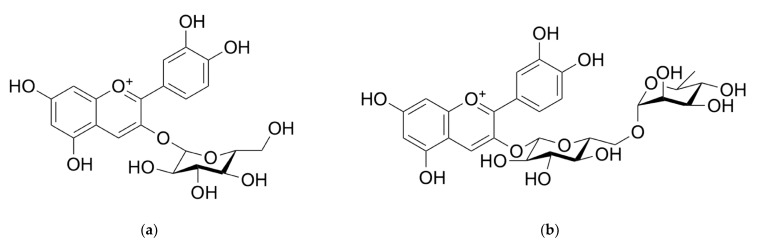

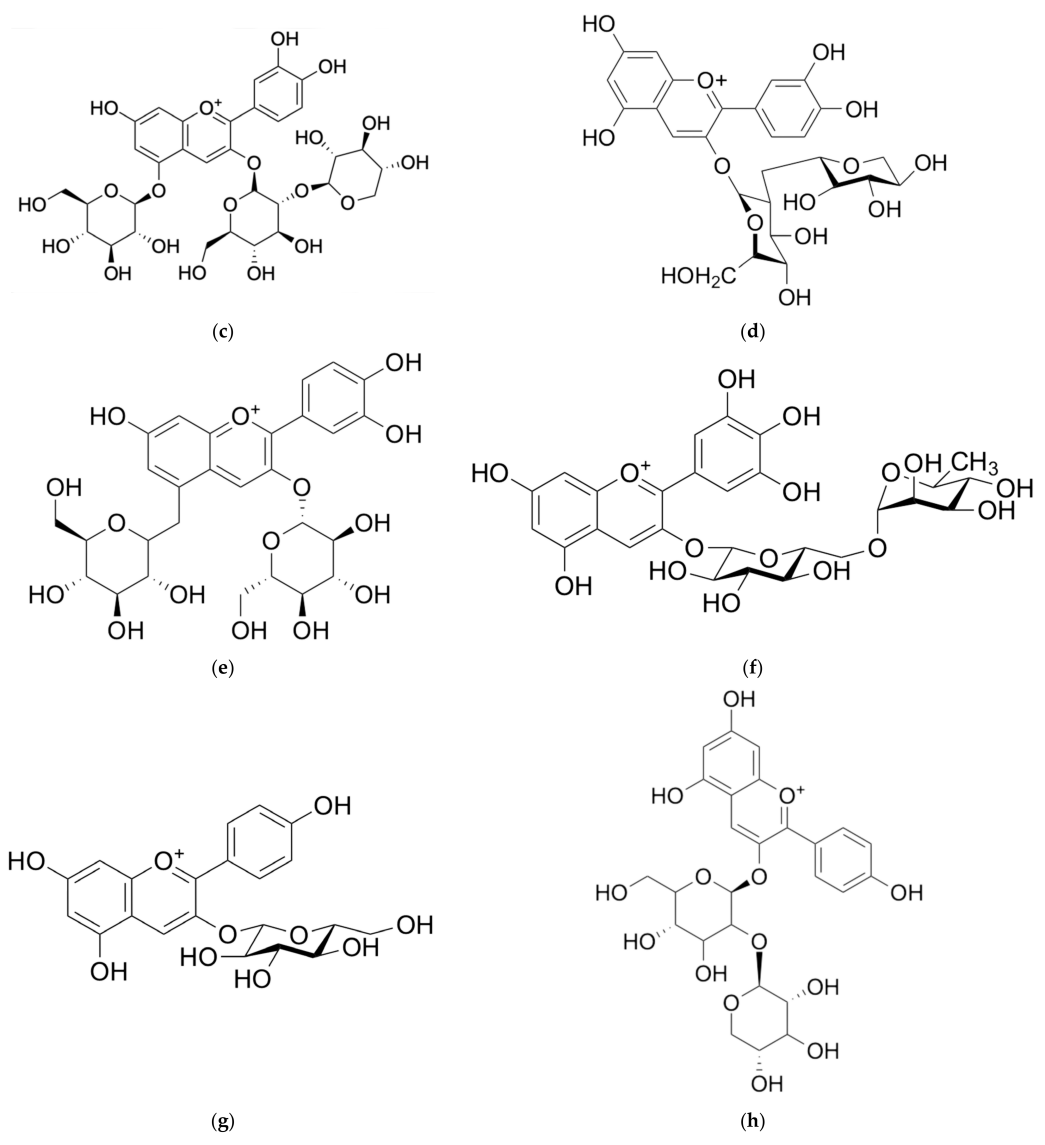
Chemical structure of anthocyanins from *Sambucus nigra* fruits: (**a**) cyanidin 3-glucoside; (**b**) cyanidin 3-rutinoside; (**c**) cyanidin 3-sambubioside-5-glucoside; (**d**) cyanidin 3-sambubioside; (**e**) cyanidin 3,5-diglucoside; (**f**) delphinidin 3-rutinoside; (**g**) pelargonidin 3-glucoside; and (**h**) pelargonidin 3-sambubioside.

**Table 1:** The chemical composition of *Sambuci Flos* [23,24,25,26].

The Group of Chemicals	Examples of Substances	Content (%)
Flavonoids	keampferol, quercetin, rutin, astragalin, isoquerlemon, hyperoside, nicotiflorin	3.0%
Phenolic acids and their glycosides	3-O-Caffeoylquinic acid, 4-O-Caffeoylquinic acid, 5-O-Caffeoylquinic acid, 1,5-Di-O-caffeoylquinic acid, 3,5-Di-O-caffeoylquinic acid, 3,4-Di-O-caffeoylquinic acid, 4,5-Di-O-caffeoylquinic acid, 3-O-p-Coumaroylquinic acid, 5-O-p-Coumaroylquinic acid, Chlorogenic acid, P-coumaric acid, Ferulic acid and their glucosides	3.0%
Triterpenes	α- and β-amyrin, ursolic acids, oleanoic acid, benzoic acid	1.0%
Sterols	β-sitosterol, campesterol, stigmasterol, cholesterol	1.0%

## Data Availability

Not applicable.
